# Examining adaptations to water stress among farming households in Sri Lanka’s dry zone

**DOI:** 10.1007/s13280-017-0904-z

**Published:** 2017-02-16

**Authors:** Nicholas E. Williams, Amanda Carrico

**Affiliations:** 397 UCB, Boulder, CO 90309 USA

**Keywords:** Agricultural adaptation, Climate change, Irrigated agriculture, Sri Lanka

## Abstract

Climate change is increasing water scarcity in Sri Lanka. Whether these changes will undermine national-level food security depends upon the ability of the small-scale farmers that dominate rice production and the institutions that support them to overcome the challenges presented by changing water availability. Analyzing household survey data, this research identifies household, institutional, and agroecological factors that influence how water-stressed farmers are working to adapt to changing conditions and how the strategies they employ impact rice yields. Paralleling studies conducted elsewhere, we identified institutional factors as particularly relevant in farmer adaptation decisions. Notably, our research identified farmers’ use of hybrid seed varietals as the only local climate adaptation strategy to positively correlate with farmers’ rice yields. These findings provide insight into additional factors pertinent to successful agricultural adaptation and offer encouraging evidence for policies that promote plant breeding and distribution in Sri Lanka as a means to buffer the food system to climate change-exacerbated drought.

## Introduction

Global food production systems are threatened by rapidly changing and increasingly unpredictable climatic conditions. The impacts of these changes are disproportionately shared and pose complex implications for the 500 million small-scale farmers that are responsible for feeding many of the world’s most vulnerable people (IPCC 2014). These farmers are often resource-poor, potentially complicating their ability to adapt to and rebound from challenging economic or weather events (Adger et al. 2003). Additionally, the crop losses or yield reductions farmers may suffer as a result of climatic instability have implications that go beyond the household, impacting regional food security and economic sovereignty (Adger et al. 2003). Therefore, there is growing concern with how best to support small-scale farmers’ adaptations to climate change and other global change processes in ways that increase household economic and food security without compromising the food supply (Adger et al. 2005; Stringer et al. 2009).

Vulnerable populations have multiple pathways for adapting to the changing climate (Davidson 2016) and these strategies are shaped by socio-ecological context (Berkes and Jolly 2002; Valdivia et al. 2010). Thus, local-level studies are critical to building an understanding of how the adaptive strategies farmers employ help to achieve food and economic security across local and regional scales. For example, livelihood shifts, such as seeking local or extra-local wage labor, are a primary means through which households can buffer themselves against the impacts of variable climatic conditions (Ellis 2000). Increasing wage labor opportunities is thus a common policy prescription for rural development planning (Dube et al. 2016). Yet, while augmenting buying power, off-farm labor’s impacts on agricultural productivity are equivocal. Local labor or the short-term migration of a household member may not necessarily compete with farming (Paavola 2008); however, in some cases, these livelihood shifts may detract from on-farm efforts (Steward 2007). Therefore, while wage labor may increase household resilience to climate change, livelihood shifts can also work to undermine regional (and potentially household-level) food security (Bryceson 1996).

To address climate change adaptation while supporting agricultural livelihoods, governments and non-governmental organizations often promote non-traditional, drought-tolerant crops as a viable way to maintain subsistence resources and support economic diversification (O’Brien et al. 2004; Morton 2007). However, non-traditional crops may be culturally undesirable and over-commitment to such crops can expose farmers to the dangers of tenuous foreign markets (Benson and Fischer 2006). Thus, like wage labor, market-oriented agricultural diversification can help to buffer households from the negative impacts of climate change, but may also undermine their political and economic sovereignty by decreasing households’ attention to traditional, locally important crops (Rosset 2011).

Farmers can also extensify or employ innovative farming strategies, such as novel chemical inputs or mechanization, to maintain the viability of locally important crops (Morton 2007). Additionally, low-input innovations, such as utilizing locally developed hybrid seeds, can help to maintain yields of traditional crops (Wassmann et al. 2009; Ceccarelli et al. 2010). Notably, asset-poor farmers often require institutional infrastructure, including credit availability and consistent extension support, to optimize novel farming strategies (Howden et al. 2007; Paavola 2008; Agrawal and Perrin 2009; Bryan et al. 2013). Therefore, smallholder farmer success under changing environmental conditions is dependent upon a multitude of factors and contexts (IPCC 2014).

To increase our understanding of climate change adaptation strategies and the trade-offs they incur, we used household survey data to characterize the household, institutional, and agroecological determinants of the various adaptive pathways employed by water-stressed farmers in Sri Lanka’s dry zone and to determine how these various strategies impact farmers’ rice yields. The dry zone is Sri Lanka’s primary rice farming region and its smallholders serve as the foundation for the nation’s rice self-sufficiency (Davis et al. 2016). The dry zone is characterized by limited farmer extensification due to secure property rights coupled to established cultivation of arable land (Dunham 1982). Because each farmer’s contribution is important to the national rice supply, government programs provide fertilizer subsidies to all farmers (Davis et al. 2016). Despite these efforts, however, farmers are challenged by an increase in climate change-related drought events (Gunda et al. 2016). Our initial site survey, conducted from 2011 to 2012, found that dry zone farming households are employing a variety of adaptive strategies to buffer against climate change impacts. These include: seeking wage labor, planting non-rice crops in paddyland, and utilizing a suite of low-input rice farming strategies.

To characterize the factors that influence the climate adaptation strategies dry zone farmers pursue and identify the most effective methods for maintaining rice yields in this increasingly drought-prone system, we: (1) compared the rice yields and adaptive pathways employed by farmers who self-identified as water-stressed with those who reported adequate water supply, (2) identified a suite of household, institutional, and agroecological factors that correlate with the diverse strategies employed by water-stressed farmers in the dry zone and, (3) determined if and how these strategies impact water-stressed farmers’ rice yields.

We hypothesized that water-stressed farmers have lower yields than farmers who receive adequate irrigation water and are also more likely to employ adaptive strategies to manage water scarcity. Among water-stressed farmers, we hypothesized: (1) farmers who lack access to irrigation water are likely to divest in rice farming and engage in off-farm labor; (2) farmers with strong relationships with agricultural extension programs are most likely to utilize the strategies being promoted by these programs, i.e., planting non-rice crops in their paddylands and employing drought-adaptive rice farming practices; (3) pursing off-farm labor signals a de-emphasis on farming and competes with rice yields; (4) farmers who have allocated their limited paddylands to non-rice crops will have lower rice yields; and (5) the various drought-adaptive rice farming strategies utilized in the region will increase farmers’ yields. Ultimately, our ability to determine the differential impacts of these various strategies has important policy implications for Sri Lanka’s dry zone.

## Materials and methods

### Site description

Sri Lanka’s central highlands buffer the island’s east and northeast regions from one of the bi-yearly monsoons, creating the ‘dry zone.’ Over thousands of years of occupation, regional inhabitants developed extensive storage and irrigation systems to use wet season water to cultivate rice during the dry season. Regional irrigation infrastructure has been expanded since the colonial period in an effort to increase domestic rice production throughout the year (Somasiri 2008). Beginning in the 1970s, families have been relocated from densely populated coastal zones and given 2.5 acres of non-transferable, newly irrigated land on which to grow rice (Moore 1989; Azmi 2007). Today, dry zone farming is largely structured by state-managed irrigation systems centered around large reservoirs that dominate the region, and rice produced here helps to provide Sri Lankans with nearly half of the calories they consume (Department of Agriculture 2006). These large-scale, state-managed systems (*major systems*) exist alongside more traditional, small-scale reservoirs (*minor systems*) that typically irrigate less than 40 acres of command area. In these minor systems, decision-making authority regarding when to release water and at what frequency rests with the local farmer organization (Begum 1987; Shah et al. 2013).

Droughts are responsible for much of the crop loss that occurs in the dry zone (Disaster Information Management System 2012). While irrigation systems help to buffer many farmers from acute rainfall shortages, prolonged droughts reduce reservoir capacity and compromise the systems’ abilities to supply sufficient water to all farmers. Farmers located in large-scale systems tend to benefit from large storage capacity and a dense network of canals that divert water from the Mahaweli River in the southeastern highlands to agricultural land in the central, north, and east. Yet, for a variety of environmental and political reasons, some state-managed reservoirs receive minimal inflows from the Mahaweli River and are largely dependent on local rainfall and therefore vulnerable to climate variability. Others located at the tail-end of the Mahaweli River or main branch canals often feel the effects of intensive water withdrawals upstream and deteriorating water quality, resulting in uncertain water supplies and lower yields (Jayewardene 1990; Kumari et al. 2011). Likewise, the location and characteristics of a farmer’s land within state- or locally managed irrigation systems can result impact their irrigation water access. This is particularly true of farmers who cultivate land at the tail-end of the field-canal, who are less likely to receive adequate water during dry seasons in some systems (Guerra et al. 1998; Shah et al. 2013). Additionally, some farmers rely solely on rainfall for rice production, which limits their cultivation to the monsoon season and leaves them particularly vulnerable to the effects of water stress (World Food Programme 2007).

In an attempt to improve water use efficiency, reduce overall demand on irrigation systems, and maintain the viability of the national food supply, the Sri Lankan government is working in coordination with local farmer organizations to promote a variety of strategies. These methods include *kakulama*, a local practice of dry seeding historically utilized during the dry season; the ‘parachute method’ of broadcasting nursery-raised seedlings into muddy fields; recycling irrigation water by pumping it back to the head of a local system; and breeding and distributing hybrid seeds, particularly those that come to maturity more quickly than traditionally planted varieties (Wassmann et al. 2009). Farmers are also encouraged during drought periods to plant ‘other field crops’ that require less water than rice paddy—such as millet, maize, or soy—in lowland that is traditionally used for rice cultivation.

### Sampling design

**Fig. 1:**
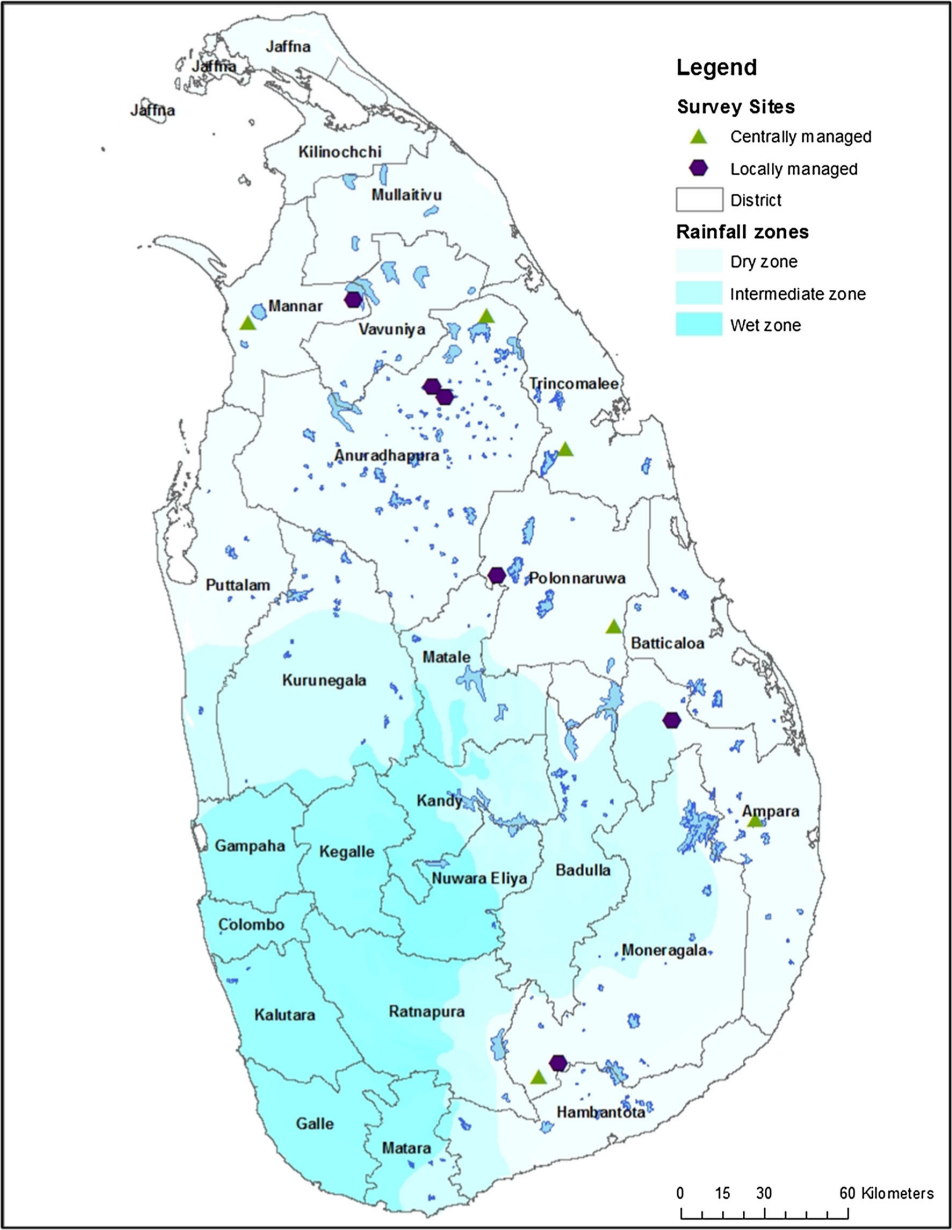
Map of survey sites

Data come from household surveys, which were collected in thirteen villages in Sri Lanka’s dry zone in May and June 2015 (Fig. 1). We used stratified random sampling to select 30 *Grama Niladhari* (*GN*) divisions, the smallest administrative unit in Sri Lanka that typically comprises between 100 and 500 households living within 1–3 villages. Because of the potential regional variability in climate change impacts (Gunda et al. 2016), the population of GN divisions was separated into three categories based on geographical location (i.e., North, North-Central, and Southeast). Weighting selection by sub-region size, eight to ten GN divisions were selected from each sub-region (*N* = 30); half of the GNs were selected from state-managed irrigation systems and half were not. Although our full sample comprises 30 communities, the data presented here come from the second phase of data collection, comprising 13 communities.

After GN divisions were selected and the villages within the GN were identified, we randomly selected one village within each GN to serve as the research site. Within each village a sampling frame was developed based on registered voter lists. If a GN did not have a single community larger than 50 households and had more than one village, households from a nearby village that shared a farmer organization were included in the sampling frame. Farmer organization leaders ensured that households on the voter lists were correctly identified rice farmers; if not, their names were removed. Thirty to eighty households (weighted for community population) were randomly selected from these lists of rice farmers in each community.

### Data collection

Results of a pilot survey developed with local agricultural specialists were used to build the survey instrument employed in this study. Trained local interviewers administered three-part surveys (less than 1 h for each session) in May and June 2015 to amenable household heads and their spouses in these 13 communities (*N* = 607) to collect demographic and household economic information that previous research suggests may impact agricultural decision-making (The World Bank 2000; World Food Programme 2007; Bangladesh Center for Advanced Studies 2011), and details about households’ farming practices. Surveys were conducted in the household’s primary language, either Sinhala or Tamil, and administered by native speakers. While politically sensitive information was not collected by our survey, native speakers were employed to lessen the influence of residual ethnic-tensions resulting from Sri Lanka’s recently ended thirty year civil war between the country’s Tamil minority and Sinhalese majority. Three potential respondents either refused or were unable to participate for a response rate of 99.5%.

### Identifying water-stress among dry zone farmers

While increased drought severity is affecting the entire region (Disaster Information Management System 2012; Gunda et al. 2016), access to irrigation water is expected to negatively correlate with farmer climate vulnerability and the need for adaptive farming strategies. Because irrigation water is used to mitigate climatic variability’s impacts on yields, our survey asked farmers about their levels of satisfaction with the irrigation water they received throughout the previous rice farming season (October 2014–February 2015). We consider farmers who reported to have not received adequate water and those who have no access to irrigation water to be water-stressed.

One survey community suffered from a reservoir collapse prior to the season. We excluded this community from analyses due to their lack of intra-community variation regarding yields. Of the 567 farmers in 12 communities remaining in our survey cohort, we identified 190 water-stressed farmers. To ensure the validity of this categorization, we compared the water-stressed with the water secure group by assessing how many seasons in the previous five years a farmer reported receiving lower than expected yields due to drought. A point bi-serial correlation showed a positive relationship (0.14) and a significant difference in the average number of seasons these farmers experienced low yields (*µ*_Water secure_ = 17.6% of seasons ± 0.01, *µ*_Water-stressed_ = 23.5% ± 0.01, *t*(362.2) = −3.5, *p* < 0.001).

We then developed a series of hierarchical random intercept models (treating community as a random factor) to determine if water stress differently impacts farmers’ adaptive behaviors and rice yields. Binomial logit models were used to determine if water-stressed farmers are differently (1) pursuing off-farm labor, (2) planting non-rice crops in paddyland, or utilizing one of the suites of water-saving agricultural practices: (3) kakulama, (4) recycling irrigation water, (5) short duration seed varietals, (6) or the parachute method than water secure farmers in the dry zone. A generalized linear model determined if water stress impacts a farmer’s seasonal yield, reported in rice bushels per acre sowed. A single statistical outlier, determined through modified *z*-scores (Iglewicz and Hoaglin 1993), was excluded from the latter analysis.

Subsequent analyses focused on the subsample of our survey cohort identified as water-stressed. Following the protocol used by Comstock et al. (2010), we developed two series of hierarchical models with random intercepts (12 communities ranging from 3 to 32 households per community; *µ* = 14.62 households) to identify (1) the factors correlated with water-stressed farmers’ employment of the agricultural adaptation strategies observed in the region and (2) how these strategies affect rice yields. This method enabled us to identify both primary and secondary factors influencing farmers’ strategies and yields.

We constructed three preliminary binomial logit models for each of our six dependent variables to understand the relationship between various household-level factors and the adaptive strategies a household employs. The first set of models included covariates relating to household demography, including: primary farmer age, their gender, household wealth (developed using principle component analysis of various material assets (Vyas and Kumaranayake 2006) and standardized to the grand mean), the number of household members assisting in farming, and the highest education level achieved by the farmer. The second set of models included institutional covariates: farmer organization (FO) participation, having attended drought outreach meeting, having received information about drought through another means, having previously contacted the national agricultural advisory service, and the proportion of a farmer’s paddyland situated in a major irrigation system. A third set of models included agroecological covariates: the size of their paddy holdings (acres), the proportion of a farmer’s paddyland that lacks connection to irrigation infrastructure (i.e., is rainfed), whether a farmer has a private agrowell, and the proportion of a farmer’s land that they self-reported as being situated at the tail-end (versus head or middle) of the canal (identified using a diagram). Final models for each dependent variable included all factors in the three preliminary model sets that were significant at the *α* ≤ 0.05 level.

We followed a similar protocol employing hierarchical linear models with random intercepts to determine whether farmers’ employment of the different adaptive strategies affects their rice yields. First, we constructed a preliminary model that included each of the drought adaptation strategies employed by dry zone farmers to determine if and how these strategies may impact a farmer’s yield. These strategies are as follows: (1) off-farm labor, (2) planting non-traditional crops in paddyland; (3) employing *kakulama*, (4) recycling irrigation water, (5) planting short duration seed varietals, or (6) using the parachute method during the 2014–2015 growing season. We then constructed three additional preliminary models including the household demographic, institutional, and agroecological covariates listed above to identify other potential predictors of yields. Our final model included each strategy and factor from the four preliminary models that were significant predictors of yields at the *α* ≤ 0.05 level to identify the most influential factors impacting yields. No outliers were identified in these models, and all analyses were conducted in R (R Core Team 2013), using the packages lme4 (Bates et al. 2015) and ggplot2 (Wickham 2009).

## Results

### Water-stressed farmers as compared to those receiving adequate irrigation water

The impact of water stress on the adaptive pathways pursued by farmers in the dry zone and their subsequent rice yields was assessed (*n* = 567, 190 water-stressed/377 water secure, in 12 communities). Water-stressed farmers experience significantly lower rice yields than farmers who were satisfied with their irrigation water (*µ*_Water secure_ = 84.60 ± 1.87 bushels, *µ*_Water-stressed_ = 80.86 ± 3.01 bushels, *p* = 0.02). We identified no difference in off-farm labor engagement or employment of any of the adaptive rice farming practices (kakulama, recycling irrigation water, short duration seed use, or the parachute method) among water-stressed versus water secure farmers. Water-stressed farmers were, however, significantly more likely to plant non-rice crops in their paddylands than water secure farmers (*p* = 0.02).

### Descriptive information regarding water-stressed farmers

Descriptive statistics for each of the variables included in our multilevel analyses are presented in Table 1 (*n* = 190 water-stressed farmers across 12 communities). Notably, 80% of farmers engaged in off-farm labor, 9% planted non-traditional crops in their paddy, and 53% employed a water conservation strategy for rice farming. Because only 4 farmers in our water-stressed subsample described using the parachute method, this method was not included in subsequent analyses.

**Table 1:** Household characteristics (*n* = 190 in 12 communities)

Variable type	*N* (%)	Median	Mean	SD	Min	Max
Demographic						
Farmer age		48	48.81	11.16	24	80
Gender (male)	171 (90)					
Wealth		0.35	0.06	0.88	−2.46	1.3
Household members assisting in farming		2	2.43	1.05	1	7
Education level						
No school	9 (5)					
Grade 1–5	43 (23)					
Grade 6–11	94 (49)					
Passed GCE OL	30 (16)					
Grade 12–13	8 (4)					
Passed GCE AL	4 (2)					
University level	2 (1)					
Institutional						
FO participation	122 (64)					
Attended drought meeting	66 (35)					
Received drought information	92 (48)					
Contacted ag. advisory service	17 (9)					
Proportion of paddyland in major irrigation system		0	33.02	46.76	0	100
Agroecological						
Total paddy holdings		2	2.97	2.94	0	18
Presence of agrowell	11 (6)					
Proportion of paddyland at tail-end of canal		0	28.07	42.12	0	100
Proportion of paddyland that is rainfed		0	8.53	26.81	0	100
Adaptive behaviors						
Off-farm labor	153 (80)					
Non-traditional crops	17 (9)					
Kakulama	33 (17)					
Recycling irrigation water	33 (17)					
Short duration seeds	52 (27)					
Parachute method	4 (2)					
Any water-saving strategy	100 (53)^a^					

^a^Percent reported is not cumulative, as farmers can employ more than one strategy in a given season

### Factors influencing farmers’ adaptive pathway use

Table 2 presents the regression coefficients and results from the models used to identify the factors that influence water-stressed farmers’ adaptive pathways. Participation in farmer organizations and high proportion paddyland at the tail-end of a canal are negatively correlated with engagement in off-farm labor. Additionally, having received less formal education and having attended drought outreach workshops significantly correlate with a farmer’s planting non-rice crops in their paddyland.

**Table 2:** Final hierarchical models of drought adaptation strategies (*n* = 190 in 12 communities)

Variable type	Off-farm labor	Non-traditional crops	Kakulama	Recycling irrigation water	Short duration seeds
Demographic					
Farmer age					
Gender (male)					
Wealth					
Household members assisting in farming					
Education level		−1.04**			
Institutional					
FO participation	−0.88*				
Attended drought meeting		1.28*			
Received drought information					0.66*
Contacted ag. advisory service					
Proportion of paddyland in major irrigation system			−0.02*	0.01*	
Agroecological					
Total paddy holdings					
Presence of agrowell					
Proportion of paddyland at tail-end of canal	−0.008***				
Proportion of paddyland that is rainfed					
Intercept	2.33	−1.78	−1.67	−3.17	0.66

* *p* ≤ 0.05, ** *p* ≤ 0.01, *** *p* ≤ 0.001

Water conservation strategies that focus on rice production each are predicted by only a single institutional or agroecological factor. Kakulama tends to be practiced by farmers whose paddylands are not fed by the state-managed irrigation systems, while recycling irrigation water is implemented by farmers whose paddylands are fed by these systems. Finally, farmers who received drought information tended to plant short duration seed varietals.

### Factors influencing rice yield

To test the factors influencing farmers’ rice yields, we included each of the strategies that dry zone farmers employ to lessen the effects of water stress (Table 3). Our preliminary model that included only the adaptive strategies showed that the planting of short duration seed varietals had a significantly positive impact on a farmer’s yield relative to not using these varietals (*β* = 10.8, *p* = 0.07). Additional preliminary models identified farmer age, use of agricultural advisory service, the proportion of a farmer’s paddyland that is rainfed, and the presence of an agrowell as factors that significantly impact rice yields. Including all significant predictors from preliminary models in a final model, our analysis shows that the proportion of a farmer’s paddyland that is rainfed had a dominant influence on their yield outcomes, eclipsing the impacts identified in preliminary models of short duration seeds, a farmer’s age, their use of the national agricultural advisory service, or their access to an agrowell (Table 3).

**Table 3:** Final model of rice yields (*n* = 190 in 12 communities)

Variable type	Yields
Adaptive behaviors	
Short duration seeds	
Covariates	
Farmer age	
Contacted ag. advisory service	
Proportion of paddyland that is rainfed	−0.27**
Presence of agrowell	
Intercept	107.90

* *p* ≤ 0.05, ** *p* ≤ 0.01, *** *p* ≤ 0.001

## Discussion

Climate change poses threats to the resource-poor farmers that are the foundation of local and regional food systems that feed many of the world’s most vulnerable populations (IPCC 2014). Therefore, identifying how best to support farmer adaptation to changing climatic conditions in ways that do not negatively impact food security is a pressing concern globally (Adger et al. 2005; Stringer et al. 2009). Our analyses provide insight into household, institutional, and agroecological factors that shape the ways in which water-stressed farmers are attempting to cope with climate change-related drought in Sri Lanka’s dry zone and how these adaptive strategies impact yields of the region’s most important crop, rice. Similar to climate adaptation studies conducted elsewhere (Agrawal 2008), we recognized farmers’ connections to formal institutions—in particular state-managed irrigation systems and agricultural extension programs—as critical to farmer adaptation to sustained water scarcity. Notably, we identified the planting of hybrid seeds varietals as particularly effective at maintaining rice yields for water-stressed farmers. These findings have implications for structuring policy in Sri Lanka and other regions where small-scale farmers are working to mitigate climate change impacts.

We hypothesized that water-stressed farmers have lower yields than farmers who receive adequate water through state- and locally managed irrigation systems and that water insecurity drives farmers to employ drought adaptation strategies. While we found significantly lower yields among self-identified water-stressed farmers, the only adaptive strategy water-stressed farmers employed more than water secure farmers was to plant non-rice crops in their paddylands, such as a drought-tolerant grain, like millet. They were no more likely to engage in off-farm labor or low-input adaptive rice farming practices than water secure farmers.

Because of the increasing severity of recent droughts (Gunda et al. 2016), farmers who have historically received adequate water may not be confident that irrigation systems will provide sufficient quantities in the coming season and therefore utilize many of the same adaptive strategies as currently water-stressed farmers. Alternatively, as previous climate adaptation research would suggest (Mertz et al. 2009; Bryan et al. 2013), dry zone farmers may in fact not be responding to climate change-related drought, but rather other shared risks, such as political or market instability that were unaccounted for in our models. Such factors may be particularly influential in driving the majority of farmers in our sample to engage in off-farm labor and should be targeted by future studies.

Focusing more narrowly on water-stressed farmers, we hypothesized that those farmers whose fields are predominantly rainfed—arguably the region’s most water-stressed—engage in more in off-farm labor than farmers with access to irrigation water. Further, we predicted a negative relationship between engagement in off-farm labor and a farmer’s rice yields. However, in parallel with our findings regarding off-farm labor among dry zone farmers more generally, we did not find off-farm labor to be more common among rainfed farmers, nor did we find off-farm labor significantly impact rice yields. We did find that off-farm labor negatively correlates with farmer organization participation. Therefore, while those seeking off-farm labor may avoid engaging in community-level farming politics, as indicated by their lack of farmer organization participation, these farmers are not disinvested in rice farming. Similar to findings in Tanzania (Paavola 2008), off-farm labor in Sri Lanka’s dry zone increases economic security, which can buffer households from climate change-related hazards and other risks, while not appearing to compromise households’ abilities to maintain farming livelihoods. In this way, encouraging off-farm labor opportunities for dry zone farmers may be a useful way to maintain both household- and regional-level stability through drought conditions.

We also identified that the proportion of a farmer’s paddyland at the tail-end of a distribution system negatively correlates with off-farm labor. Despite having controlled for wealth in our model, this effect may reflect the social status of tail-end farmers. Social inequalities are known to relate to farmers’ locations along the canal, with more powerful members of the community often occupying land closest to the reservoir (Uphoff and Wijayaratna 2000). In rare cases, these are Tamil ethnic-minority farmers living in dominantly Sinhalese communities. However, because of the tendency toward ethnic homogeneity in dry zone communities and the nature of the hierarchical analyses we employed in our study, our survey did not capture sufficient intra-community ethnic variation to determine the role that ethnic dynamics play in structuring water access. Regardless, the fact that tail-end farmers have less diversified incomes is significant, as Kumari et al. (2011) found that tail-end farmer with no off-farm income are among the most impoverished and vulnerable in Sri Lanka. Therefore, we encourage future studies to determine how social marginality impacts labor opportunities for tail-end farmers and the role of ethnic politics in Sri Lanka on irrigation water access within (and between) communities.

We also hypothesized that farmers who have relationships with agricultural extension programs would be more likely to manage drought conditions by planting non-rice crops in their paddylands and employing low-input adaptive rice farming strategies. In addition to being practiced by farmers with the least formal education, planting non-rice crops positively correlates with having attended outreach workshops focusing on drought, and the use of short duration seed varietals relates to having formally received information about drought from a (non-)governmental organization. Together these findings suggest that farmers may be receptive to the information they are receiving and that outreach efforts of this sort may be worthwhile. We also found that recycling irrigation water and *kakulama* are positively and negatively correlated, respectively, with a farmer’s connection with state-managed irrigation systems. These findings, which are consistent with previous research conducted in Sri Lanka and elsewhere (Uphoff and Wijayaratna 2000; Agrawal 2008; Agrawal and Perrin 2009; Gedara et al. 2012), reflect the central role that formal (and informal) institutions play in agricultural climate adaptation.

Finally, we constructed a series of models which enabled us to detect both primary and secondary factors influencing rice yields among water-stressed farmers. We hypothesized that planting non-rice crops in paddyland results in a trade-off with rice yield, while the low-input rice farming strategies utilized in the region increase yields. Interestingly, however, initial models revealed that like off-farm labor, planting non-traditional crop did not impact rice yields. The only climate adaptation strategy that impacted rice yields (positively or negatively) was short duration hybrid seeds use. Yet, the significance of this adaptive strategy was diminished in our final model by the negative impact of rainfed irrigation on yields.

These results highlight the critical role of Sri Lanka’s historical irrigation systems for supporting rice farming in the country’s dry zone. Being unable to receive controlled inflows of water throughout the farming season is detrimental to farmers’ yields. However, the positive impact of planting hybrid seed varietals on rice yields across communities provides insight into the types of adaptive behaviors that are a key to farmers’ successful climate change adaptation. These hybridized varietals, developed by national and international plant breeding centers, do not require farmers to alter their farming strategies, but simply to plant rice that comes to seed more quickly than traditionally planted cultivars (Dhanapala 2006). Thus, while the development and distribution of these seeds requires institutional involvement, planting hybrid seeds demands less coordination for execution than the other climate adaptation strategies being promoted by extension programs (i.e., *kakulama*, recycling irrigation water, and the parachute method). Even in these long-established and well-coordinated irrigation systems, system- and local-level changes pose both logistical hurdles and the potential exclusion of certain farmers. Therefore, supporting adaptations that can be managed at the farm-level may be the most viable option for rapid climate change adaptation in Sri Lankan rice farming and elsewhere.

## Conclusion

The state-level institutions that both provide irrigation water and help to develop and promote drought-adaptive farming strategies play a critical role in farmers’ adaptation to climate change in Sri Lanka’s dry zone. Echoing prior research conducted elsewhere, our study shows that farmers without formal institutional (particularly infrastructural) support are the most vulnerable to climate change, and we encourage that climate adaptation efforts target these populations, possibly working to create wage labor opportunities particularly for those farmers in areas where irrigation system expansion is challenging. Further, while each of the adaptive strategies employed by water-stressed farmers may be helping to maintain rice yields that would be otherwise reduced by drought, planting hybrid seed varietals appears to be the most effective strategy for maintaining yields in the face of water stress. While these hybrid seeds may reduce farmers’ yields in optimal conditions (Dhanapala 2006; Wassmann et al. 2009), they are more effective than traditional varietals under drought conditions. Additionally, planting short duration seed varietals requires less coordination and institutional intervention than the other climate change adaptation strategies being promoted in the dry zone, underscoring the importance and challenges of state-level institutions’ involvement in climate change adaptation. Ultimately, the positive effect of short duration seeds on water-stressed farmers yields suggest that the continued and increased promotion of these seeds may be the most effective method for maintaining Sri Lanka’s rice supply as climate change-driven drought risk intensifies.

## References

[CR1] Adger WN, Arnell NW, Tompkins EL (2005). Successful adaptation to climate change across scales. Global Environmental Change.

[CR2] Adger WN, Huq S, Brown K, Conway D, Hulme M (2003). Adaptation to climate change in the developing world. Progress in Development Studies.

[CR3] Agrawal, A. 2008. The role of local institutions in adaptation to climate change. *Social Development*, 734–764. International Forestry Research and Institutions Program.

[CR4] Agrawal, A., and N. Perrin. 2009. Climate adaptation, local institutions and rural livelihoods. In *Adapting to climate change: Thresholds, values, governance*, eds. W.N. Adger, L. Lorenzoni, and K. O’Brien, 350–67. School of Natural Resources and Environment, University of Michigan, International Forestry Resources and Institutions Program, IFRI, Working Paper, W081-6.

[CR5] Azmi F (2007). Changing livelihoods among the second and third generations of settlers in system H of the accelerated Mahaweli development project (AMDP) in Sri Lanka. Norwegian Journal of Geography.

[CR6] Bangladesh Center for Advanced Studies. 2011. *Summary of baseline household survey results: Paikgacha, Khulna (Block*- *3), Bangladesh*.

[CR7] Bates D, Mächler M, Bolker B, Walker S (2015). Fitting linear mixed-effects models using lme4. Journal of Statistical Software.

[CR8] Begum S (1987). minor tank water management in the dry zone of Sri Lanka.

[CR9] Benson P, Fischer EF (2006). Broccoli and desire: global connections and Mayan struggles in postwar Guatemala.

[CR10] Berkes F, Jolly D (2002). Adapting to climate change: social-ecological resilience in a Canadian western arctic community. Ecology and Society.

[CR11] Bryan E, Ringler C, Okoba B, Roncoli C, Silvestri S, Herrero M (2013). Adapting agriculture to climate change in Kenya: household strategies and determinants. Journal of Environmental Management.

[CR12] Bryceson DF (1996). Deagrarianization and rural employment in sub-Saharan Africa: a sectoral perspective. World Development.

[CR13] Ceccarelli S, Grando S, Maatougui M, Michel M, Slash M, Haghparast R, Rahmanian M, Taheri A (2010). Plant breeding and climate changes. The Journal of Agricultural Science.

[CR14] Comstock N, Miriam Dickinson L, Marshall JA, Soobader M-J, Turbin MS, Buchenau M, Litt JS (2010). Neighborhood attachment and its correlates: exploring neighborhood conditions, collective efficacy, and gardening. Journal of Environmental Psychology.

[CR15] Davidson D (2016). Gaps in agricultural climate adaptation research. Nature Climate Change.

[CR16] Davis KF, Gephart JA, Gunda T (2016). Sustaining food self-sufficiency of a nation: The case of Sri Lankan rice production and related water and fertilizer demands. Ambio.

[CR17] Department of Agriculture (2006). Crop recommendations.

[CR18] Dhanapala, M.P. 2006. *Bridging the rice yield gap in Sri Lanka*. Bangkok: United Nations Food and Agriculture Organization (UNFAO).

[CR19] Disaster Information Management System. 2012. *Disaster event & impact profile*. Sri Lanka National report on disaster risk, poverty and human development relationship, Colombo.

[CR20] Dube T, Moyo P, Ncube M, Nyathi D (2016). The impact of climate change on agro-ecological based livelihoods in Africa: a review. Journal of Sustainable Development.

[CR21] Dunham DM (1982). Politics and land settlement schemes: the case of Sri Lanka. Development and Change.

[CR22] Ellis F (2000). Rural livelihoods and diversity in developing countries.

[CR23] Gedara KM, Wilson C, Pascoe S, Robinson T (2012). Factors affecting technical efficiency of rice farmers in village reservoir irrigation systems of Sri Lanka. Journal of Agricultural Economics.

[CR24] Guerra LC, Bhuiyan SII, Tuong TPP, Barker R (1998). Producing more rice with less water from irrigated systems.

[CR25] Gunda T, Hornberger GM, Gilligan JM (2016). Spatiotemporal patterns of agricultural drought in Sri Lanka: 1881–2010. International Journal of Climatology.

[CR26] Howden SM, Soussana J-F, Tubiello FN, Chhetri N, Dunlop M, Meinke H (2007). Adapting agriculture to climate change. Proceedings of the National Academy of Sciences of the United States of America.

[CR27] Iglewicz B, Hoaglin DC, Mykytka EF (1993). Volume 16: how to detect and handle outliers. The ASQC basic references in quality control: statistical techniques.

[CR28] IPCC. 2014. *Climate change 2014: synthesis report.* Contribution of Working Groups I, II and III to the fifth assessment report of the intergovernmental panel on climate change, IPCC.

[CR29] Jayewardene J, Puttaswamaiah K (1990). Economic inequality between top-enders and tail-enders in Sri Lankan irrigation schemes. Poverty and rural development: planners, peasants, and poverty.

[CR30] Kumari B, Thiruchelvam S, Dissanayake H, Lasantha T (2011). Crop diversification and income inequality in irrigation systems: the case of minipe. Tropical Agricultural Research.

[CR31] Mertz O, Mbow C, Reenberg A, Diouf A (2009). Farmers’ perceptions of climate change and agricultural adaptation strategies in rural sahel. Environmental Management.

[CR32] Moore M (1989). The ideological history of the Sri Lankan “peasantry”. Modern Asian Studies.

[CR33] Morton JF (2007). The impact of climate change on smallholder and subsistence agriculture. Proceedings of the National Academy of Sciences of the United States of America.

[CR34] O’Brien K, Leichenko R, Kelkar U, Venema H, Aandahl G, Tompkins H, Javed A, Bhadwal S (2004). Mapping vulnerability to multiple stressors: climate change and globalization in India. Global Environmental Change.

[CR35] Paavola J (2008). Livelihoods, vulnerability and adaptation to climate change in Morogoro, Tanzania. Environmental Science & Policy.

[CR36] R Core Team. 2013. R: A language and environment for statistical computing. R Foundation for Statistical Computing, Vienna, Austria. URL http://www.R-project.org/.

[CR37] Rosset P (2011). Food sovereignty and alternative paradigms to confront land grabbing and the food and climate crises. Development.

[CR38] Shah T, Samad M, Ariyaratne R, Jinapala K (2013). Ancient small-tank irrigation in Sri Lanka: continuity and change. Economic & Political Weekly.

[CR39] Somasiri HPS (2008). Participatory management in irrigation development and environmental management in Sri Lanka. Journal of Developments in Sustainable Agriculture.

[CR40] Steward A (2007). Nobody farms here anymore: livelihood diversification in the Amazonian community of Carvão, a historical perspective. Agriculture and Human Values.

[CR41] Stringer LC, Dyer JC, Reed MS, Dougill AJ, Twyman C, Mkwambisi D (2009). Adaptations to climate change, drought and desertification: local insights to enhance policy in Southern Africa. Environmental Science & Policy.

[CR42] The World Bank. 2000. *Designing household survey: questionnaires for developing countries*. ed. M. Grosh and P. Glewwe. The World Bank, Washington.

[CR43] Uphoff N, Wijayaratna CM (2000). Demonstrated benefits from social capital: the productivity of farmer organizations in Gal Oya, Sri Lanka. World Development.

[CR44] Valdivia C, Seth A, Gilles JL, García M, Jiménez E, Cusicanqui J, Navia F, Yucra E (2010). Adapting to climate change in Andean ecosystems: landscapes, capitals, and perceptions shaping rural livelihood strategies and linking knowledge systems. Annals of the Association of American Geographers.

[CR45] Vyas S, Kumaranayake L (2006). Constructing socio-economic status indices: how to use principal components analysis. Health policy and planning.

[CR46] Wassmann R, Jagadish SVK, Heuer S, Ismail A, Redona E, Serraj R, Singh RK, Howell G (2009). Climate change affecting rice production: the physiological and agronomic basis for possible adaptation strategies. Advances in Agronomy.

[CR47] Wickham, H. 2009. *Ggplot2: elegant graphics for data analysis*. New York: Springer. ISBN: 978-0-387-98140-6.

[CR48] World Food Programme (2007). Sri Lanka food security assessment, based on the integrated food security and humanitarian phase classification approach.

